# Supplementary material for: Statins Induce Locomotion and Muscular Phenotypes in Drosophila melanogaster That Are Reminiscent of Human Myopathy: Evidence for the Role of the Chloride Channel Inhibition in the Muscular Phenotypes
Source: Cells. 2022 Nov 8;11(22):3528. doi: 10.3390/cells11223528 (PMC9688544; doi:10.3390/cells11223528)
Supplement: Supplementary file 1 [file cells-11-03528-s001.zip › cells-1909014-supplementary.pdf]

Supplementary data

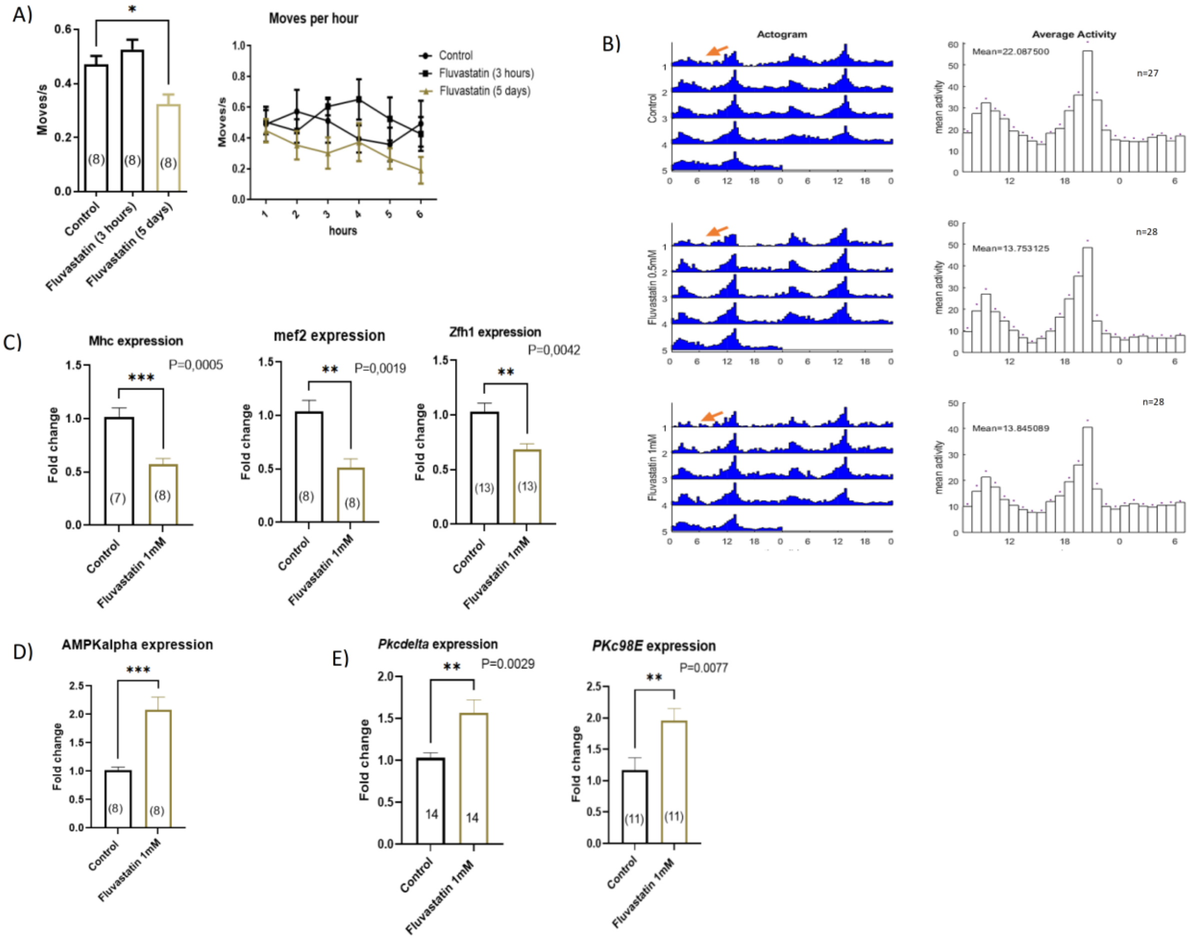

Supplementary Figure S1: A) the histogram shows the locomotion activity (the average moves) for acute (three hours) and chronic (five days) exposure to 0.5mM fluvastatin using the forced-climbing locomotion system. 1-2 days old male virgin CSOCR flies were used. The statistical difference between the three groups was calculated using one-way ANOVA with Bonferroni's multiple comparisons whereby the number of flies is indicated in brackets. B) The actogram and average activity histograms show the activity of the flies per 24 hours for five days using DAMS. C) Histograms show quantification of transcript levels *Mef2*, myocyte enhancer factor-2; *Mhc*, Myosin heavy chain; *zfh1*, Zn finger homeodomain 1; all were normalized with Rpl32 (reference gene). D) Histograms show quantification of transcript levels of *AMPKα,* AMP-activated protein kinase α subunit, normalized with Rpl32 (reference gene). E) The histograms show quantification of transcript levels, and fold change, for *Pkcdelta*, Protein kinase C δ, and *Pkc98E*, Protein C kinase 98E, normalized by the *RpL32* (reference gene). The whole body of 10 flies was used in each sample

and the number of samples is indicated in brackets. In B, C, D & E, in the fluvastatin group, flies were treated with 1mM mixed with food and in the control, flies were fed with food mixed with a corresponding volume of water. In each histogram, the bars represent the mean values ± SEM. Shapiro- Wilk test was used to check normality. Accordingly, Student's t-test or Mann-Whitney test was used to calculate statistical significance whereby **, P ≤ 0.01; *** P ≤0 .001, **** P ≤0 .0001.

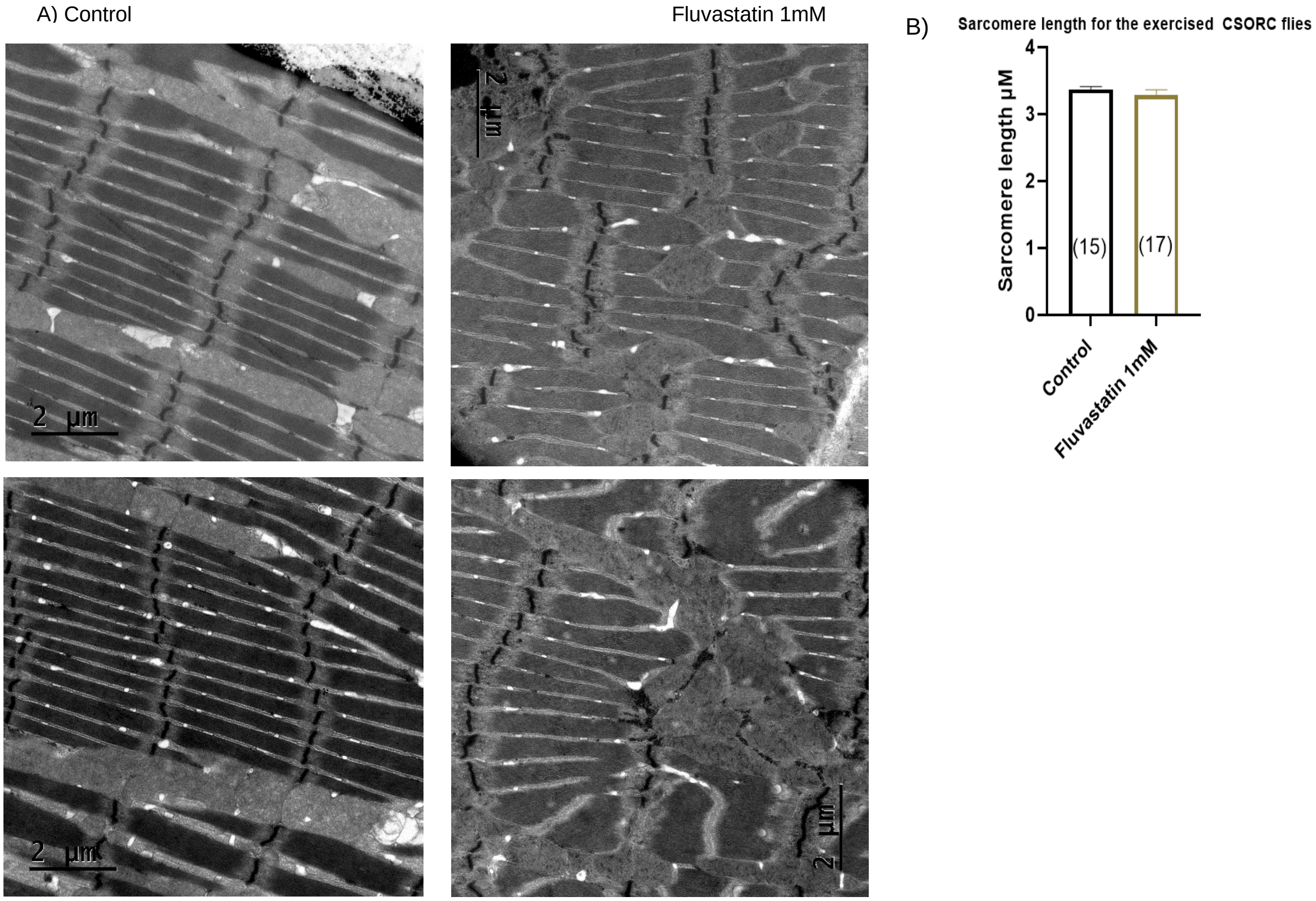

Supplementary Figure S2: A) Transmission electron microscopy micrographs for the morphology of longitudinal section of the skeletal muscles of femur segment of CSORC fly's leg. In the fluvastatin group, CSORC flies were treated with 1.0mM fluvastatin while the control group was fed with food, both for five days and the flies were exercised for six hours using the force-climbing locomotion system. B) Histogram showed the sarcomere length in µM, as shown in the image, which was measured in a number of TEM micrographs indicated in brackets. In each histogram, the bars represent the mean values ± SEM and Student's t-test was used to calculate the significance after checking the normality using the Shapiro-Wilk test.

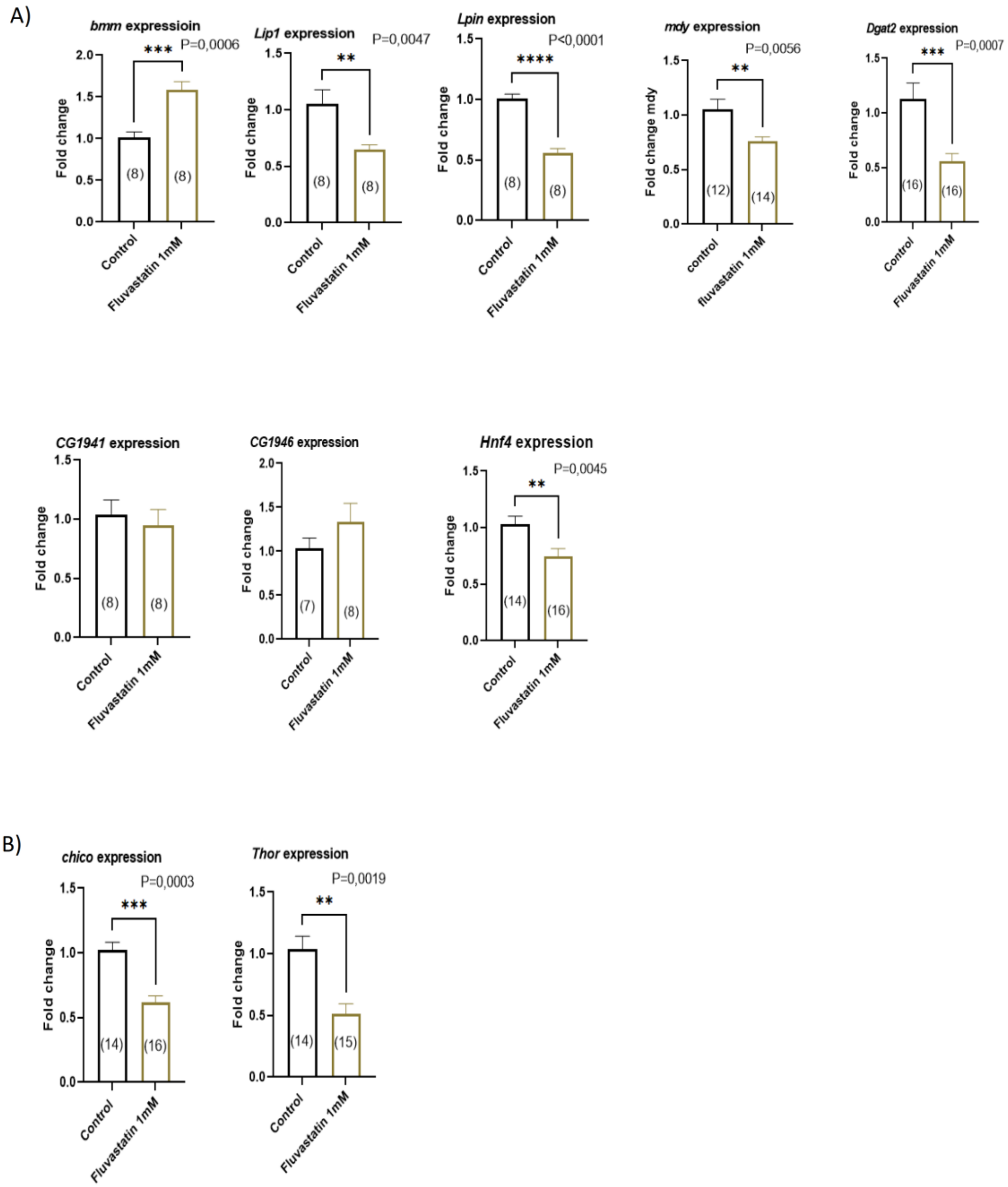
A)
bmm expressioin
P=0,0006
***
Fold change
2.0
1.5
1.0
0.5
0.0
(8)
(8)
Control
Fluvastatin 1mM
Lip1 expression
P=0,0047
**
Fold change
1.5
1.0
0.5
0.0
(8)
(8)
Control
Fluvastatin 1mM
Lpin expression
P<0,0001
****
Fold change
1.5
1.0
0.5
0.0
(8)
(8)
Control
Fluvastatin 1mM
mdy expression
P=0,0056
**
Fold change mdy
1.5
1.0
0.5
0.0
(12)
(14)
control
fluvastatin 1mM
Dgat2 expression
P=0,0007
***
Fold change
1.5
1.0
0.5
0.0
(16)
(16)
Control
Fluvastatin 1mM
CG1941 expression
Fold change
1.5
1.0
0.5
0.0
(8)
(8)
Control
Fluvastatin 1mM
CG1946 expression
Fold change
2.0
1.5
1.0
0.5
0.0
(7)
(8)
Control
Fluvastatin 1mM
Hnf4 expression
P=0,0045
**
Fold change
1.5
1.0
0.5
0.0
(14)
(16)
Control
Fluvastatin 1mM
B)
chico expression
P=0,0003
***
Fold change
1.5
1.0
0.5
0.0
(14)
(16)
Control
Fluvastatin 1mM
Thor expression
P=0,0019
**
Fold change
1.5
1.0
0.5
0.0
(14)
(15)
Control
Fluvastatin 1mM

Supplementary Figure S3: Fluvastatin treatment is associated with disruption of lipid metabolism and insulin hemostasis. A, B) Histograms show relative expression of important genes in lipid metabolism and insulin signalling, whereby *bmm*, brummer; *Lip1*, Lipase 1; *Lpin*, Lipin; *mdy*, midway; *Dgat2*, Diacylglycerol O-acyltransferase; *Hnf4*, Hepatocyte nuclear factor 4. In the fluvastatin group, flies were treated with 0.1mM whereas flies in the control group were treated with food only, both for five days. The whole body of 10 flies was used in each sample and the number of samples is indicated in brackets. In each histogram, the bars represent the mean values ± SEM. Shapiro- Wilk test was used to check normality. Student's t-test for normally distributed data while Mann-Whitney test for non-normal distributed data whereby *, P≤0.05; **, P ≤ 0.01; *** P ≤0 .001, **** P ≤0 .0001.

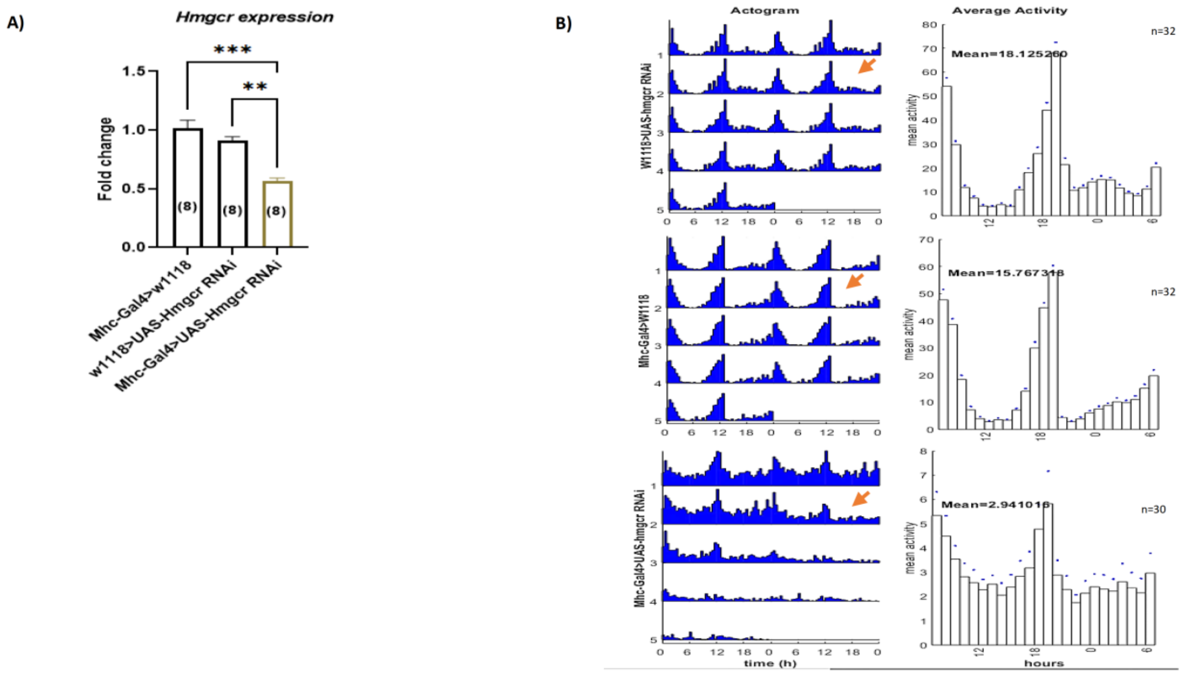

Supplementary Figure S4: *Hmgcr* knockdown in all muscles was carried out by using the Gal4-UAS system, where $Hmgcr^{RNAi}$ knockdown flies (*Mhc-Gal4>UAS-Hmgcr RNAi*) and controls (*Mhc-Gal4>w1118 and w1118>UAS-RNAi*). Male flies, taken immediately after eclosion and fed with normal food, were collected for experiments. A) The histogram shows the quantification of transcript levels, and fold change, for *Hmgcr* normalized by the *RpL32* (reference gene) where the whole body of 10 flies was used in each sample and the number of samples is indicated in the brackets. B) The right graph shows the octogram and the left shows the average activity of the flies for the five days using DAMs and n=30. Each bar represents the mean value (±SEM) and the Shapiro- Wilk test was used to check normality. The statistical difference was calculated using one-way ANOVA with Bonferroni's multiple comparisons whereby **, P ≤ 0.01, ****, P ≤ 0 .0001.

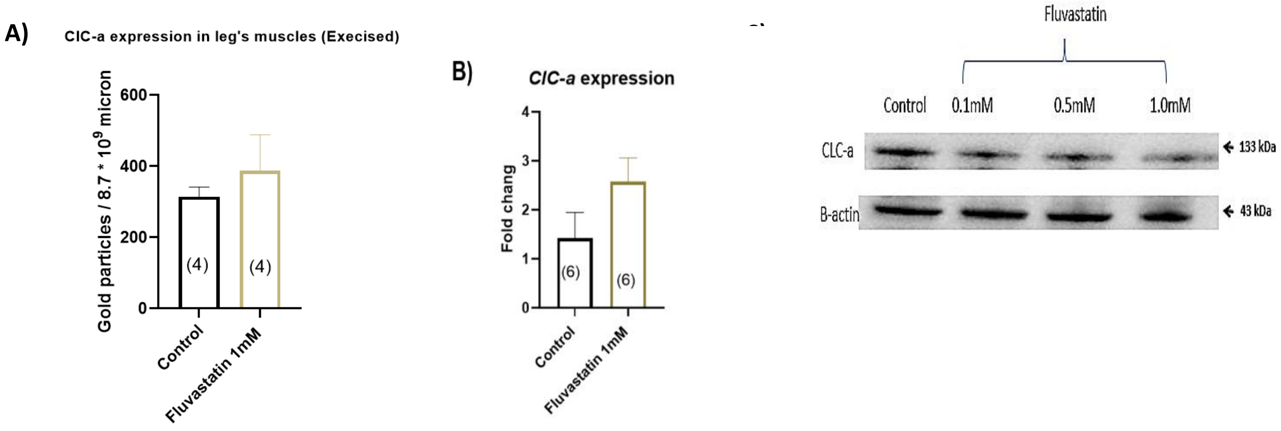

Supplementary Figure S5: A) Using ImageJ software, the histogram represents the quantification of ClC-a gold particle of dissected skeletal muscles of femur and tibia segments of CSORC flies that were exercised for 6 hours in Forced Climbing Activity system before dissection. Student's t-test was used to calculate the statistical significance and the number of TEM grids used is indicated in the brackets. B) Histograms show quantification of transcript *a,* normalized with Rpl32 (reference gene). The whole body of 10 flies was used in each sample and the number of samples is indicated in the brackets. Statistical significance was used to calculate by the Mann-Whitney test and each bar represents the mean value (±SEM). C) The blot image represents western blotting for the relative protein expression of ClC-a and β-Actin. The whole body of 10 flies was used per sample. In the fluvastatin group, flies were treated with 1mM fluvastatin mixed with food while in the control group, flies were treated with food mixed with a corresponding volume of water.

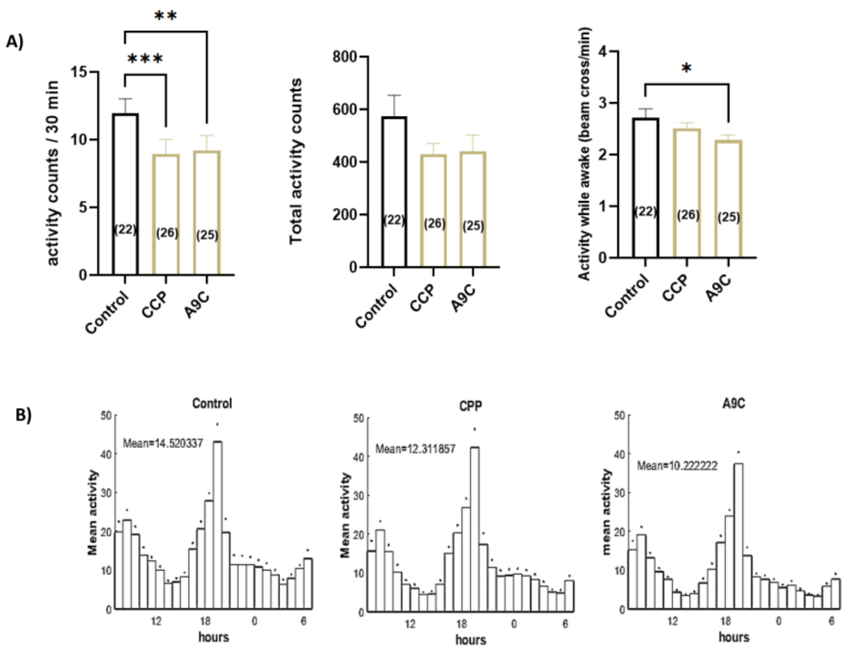

Supplementary Figure S6: Chloride channel blockers induced lower activity in CSORC flies. A) The histograms show the activity counts/30 min, total activity accounts and activity while awake A) Histogram shows the average activity of flies treated with chloride channel blockers, 1mM CPP and 1mM A9C. For the control group, flies were treated with food, all for five days. The number of flies is indicated in the brackets and one-way ANOVA with the Kruskal-Wallis test was used to calculate statistical significance whereby *, P≤0.05; **, P ≤ 0.01, ***, P ≤ 0 .001. In each histogram, the bars represent the mean values ± SEM and the number of samples is indicated in the brackets.

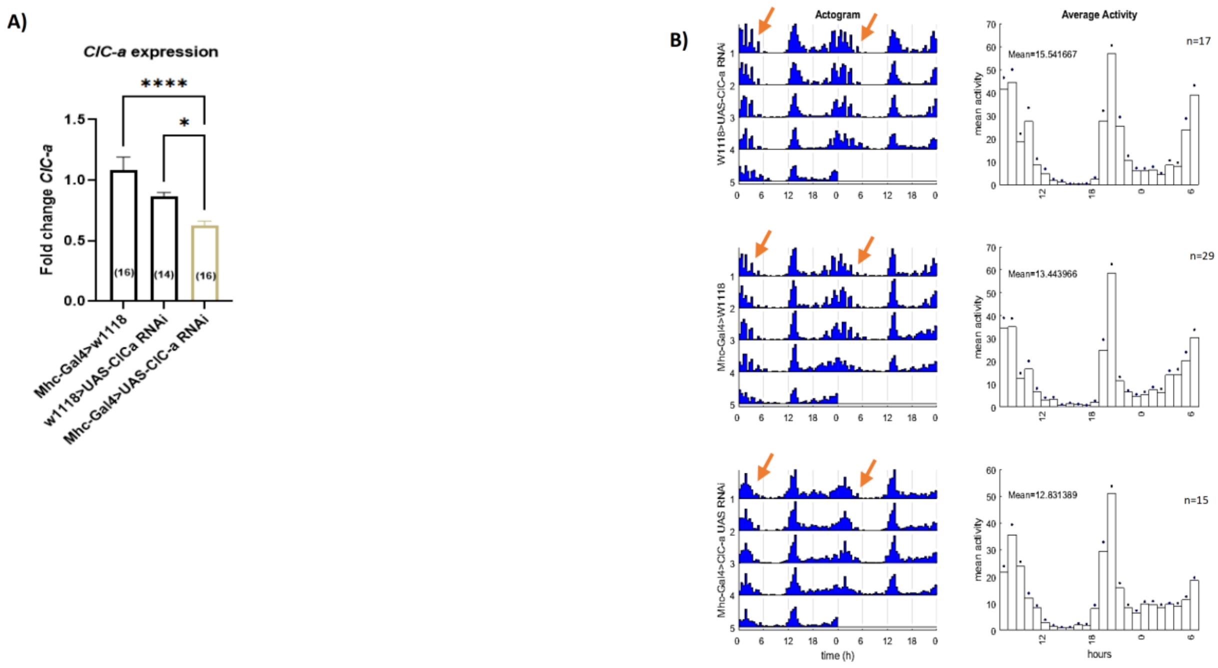

Supplementary Figure S7: *ClC-a* Knockdown was carried out using the UAS-Gal4 system where *ClC-a* ^{RNAi} knockdown flies (*Mhc-Gal4>UAS-ClC-a RNAi*) and controls (*Mhc-Gal4>w1118* and *w1118>UAS-ClC-a RNAi*). Male days after eclosion and fed with food. A) The histogram shows quantification of transcript levels, and fold change, for *ClC-a* normalized by the *RpL32* (reference gene) where the whole body of 10 flies was used in each sample and the number of samples is indicated in brackets. B) The right graph shows the octogram and the left shows the average activity of the flies for the five days using DAMs. Shapiro test was used to check normality wherein A, D &G the statistical difference was calculated using one-way ANOVA with Bonferroni's multiple comparisons for normally distributed data and Kruskal–Wallis one-way tests for otherwise data whereby *, P≤0.05; **** P ≤0 .0001.

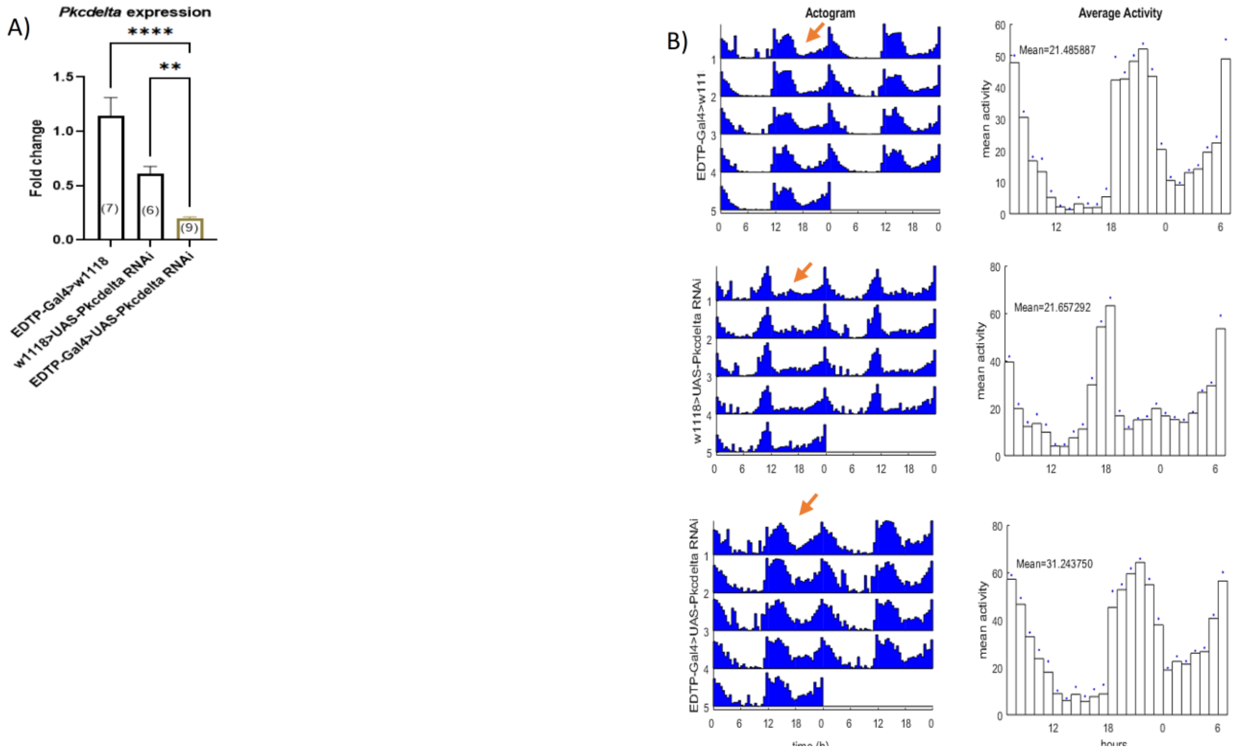

Supplementary Figure S8: Pkcdelta knockdown was carried out by using the Gal4-UAS system whereby *Pkcdelta*^{RNAi} knockdown flies (*EDTP-Gal4>UAS-Pkcdelta RNAi*) and controls (*EDTP-Gal4>w1118* and *w1118>UAS-Pkcdelta RNAi*). Male flies, aged five days after eclosion, were collected for DAMS and LAMS and fed with normal food. A) The histogram shows quantification of transcript levels, and fold change, for *Pkcdelta* normalized by the *RpL32* (reference gene) where the whole body of 10 flies per sample was used. Each bar represents the mean value (±SEM) and the number of samples is indicated in the brackets. B) The graphs show both actogram and average activity for five days using DAMS, Shapiro test was used to check the normality test. Accordingly, the statistical difference was calculated using one-way ANOVA with Bonferroni's multiple comparisons for normally distributed data and Kruskal–Wallis one-way tests for otherwise data whereby **, P ≤ 0.01, ****, P ≤ 0 .0001.

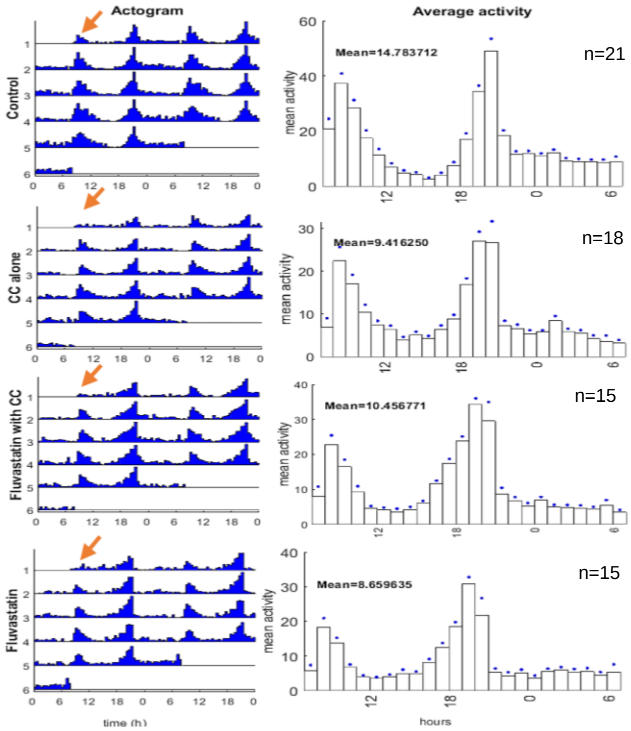

Supplementary Figure S9: Male virgin CSORC flies were used for the experiments. The left histogram shows the actogram while the right shows the average activity using DAMS, both for five days. The control group was fed with normal food alone, the fluvastatin group was fed with 0.5mM fluvastatin, and the fluvastatin & CC group was fed with both 0.5mM fluvastatin and 100μM CC, and the CC group was fed with 100μM CC.
